# Transfer RNA-derived small RNA tRF-Glu-CTC attenuates neointimal formation via inhibition of fibromodulin

**DOI:** 10.1186/s11658-023-00523-z

**Published:** 2024-01-03

**Authors:** Qi-Lan Jiang, Jia-Ying Xu, Qing-Ping Yao, Rui Jiang, Qin Xu, Bo-Tao Zhang, Tao Li, Jun Jiang

**Affiliations:** 1 Department of Clinical Nutrition, Affiliated Hospital of Southwest Medical University, Luzhou, Sichuan Province China; 2 Department of General Surgery (Thyroid Surgery), The Affiliated Hospital of Southwest Medical University, 25 Taiping Street, Jiangyang District, Luzhou, 646000 Sichuan Province China; 3 Institute of Mechanobiology and Medical Engineering, School of Life Science and Biotechnology, Shanghai Jiao Tong University, Shanghai, China; 4 Department of Urology, Affiliated Hospital of Southwest Medical University, Luzhou, Sichuan Province China; 5 Key Laboratory of Medical Electrophysiology of Ministry of Education, Collaborative Innovation Center for Prevention and Treatment of Cardiovascular Disease, Institute of Cardiovascular Research, Southwest Medical University, Luzhou, 646000 Sichuan Province China

**Keywords:** Transfer RNA (tRNA)-derived small RNAs (tsRNAs), Vascular smooth muscle cell, Vascular remodeling, Neointimal hyperplasia (NIH), Proliferation, Migration

## Abstract

Neointimal hyperplasia is a pathological vascular remodeling caused by abnormal proliferation and migration of subintimal vascular smooth muscle cells (VSMCs) following intimal injury. There is increasing evidence that tRNA-derived small RNA (tsRNA) plays an important role in vascular remodeling. The purpose of this study is to search for tsRNAs signature of neointima formation and to explore their potential functions. The balloon injury model of rat common carotid artery was replicated to induce intimal hyperplasia, and the differentially expressed tsRNAs (DE-tsRNAs) in arteries with intimal hyperplasia were screened by small RNA sequencing and tsRNA library. A total of 24 DE-tsRNAs were found in the vessels with intimal hyperplasia by small RNA sequencing. In vitro, tRF-Glu-CTC inhibited the expression of fibromodulin (FMOD) in VSMCs, which is a negative modulator of TGF-β1 activity. tRF-Glu-CTC also increased VSMC proliferation and migration. In vivo experiments showed that inhibition of tRF-Glu-CTC expression after balloon injury of rat carotid artery can reduce the neointimal area. In conclusion, tRF-Glu-CTC expression is increased after vascular injury and inhibits FMOD expression in VSMCs, which influences neointima formation. On the other hand, reducing the expression of tRF-Glu-CTC after vascular injury may be a potential approach to prevent vascular stenosis.

**Supplementary Information:**

The online version contains supplementary material available at 10.1186/s11658-023-00523-z.

## Introduction

Under the influence of specific changes in the internal and external environment, usually chronic changes in hemodynamic conditions and/or humoral factors, blood vessels respond and undergo long-term structural changes, termed vascular remodeling [1]. Mechanistically, endothelial cells, vascular smooth muscle cells (VSMCs), and adventitial fibroblasts sense signals from the internal and external environment and respond functionally and structurally through intercellular communication, resulting in changes in vascular geometry [2]. Vascular remodeling plays an essential role in the pathophysiology of cardiovascular disease. Neointimal hyperplasia (NIH) is a process in which VSMC populations increase and functionally change in the subintima of the arterial wall and is seen in atherosclerosis, pulmonary hypertension, angioplasty, vein grafts and organ transplantation, etc. [3]. Injury, inflammation, and stretch-induced phenotypic changes, proliferation, and migration of VSMCs are the underlying causes of NIH [4].

Growth factors, cytokines, and many other biomolecules such as nucleic acids have been shown to play important roles in vascular remodeling. Non-coding ribonucleic acids (ncRNAs), including microRNA (miRNA) and long non-coding RNA (lncRNA), are RNA molecules that are not translated into protein. ncRNAs have diverse biological effects on vascular remodeling in atherosclerosis, aneurysm disease and restenosis after angioplasty by regulating endothelial cell and vascular smooth muscle functions and inflammatory responses, which have great potential in the diagnosis and the treatment of cardiovascular diseases [5–8]. Transfer RNA (tRNA)-derived small RNAs (tsRNAs) or so-called tRNA-derived fragments (tRFs) are a novel class of non-coding RNAs that are typically generated from mature or precursor tRNAs by endonuclease cleavage under stress conditions [9]. Based on different cleavage positions, tsRNAs are classified into subtypes such as tRF-1, tRF-2, tRF-3, tRF-5, i-tRF, 5'tiRNA, and 3'tiRNA [10]. tsRNAs can share similarities with the canonical miRNA pathway, or participate in reverse transcription as retroelements, or interact with proteins to regulate gene expression or translation. They have been implicated in many life processes and diseases, including regulation of cellular homeostasis, stress adaptation, metabolic diseases, aging, reproduction, stress, and organ damage etc., and have been found to have potential use as biomarkers or therapeutic targets [11, 12].

tsRNAs have been implicated in the pathogenesis of a variety of cardiovascular diseases, including atherosclerosis, myocardial infarction, pulmonary hypertension, cardiac hypertrophy, and heart failure [13–16]. In a previous study, we found that tRNA-Gln-CTG-derived fragment (tRF-Gln-CTG) was highly expressed in balloon-injured rat carotid arteries and promoted VSMC proliferation by negatively regulating the expression of Fas cell surface death receptor (FAS) [17]. With the continuous updating and improvement of analysis methods and tsRNA databases, especially the emergence of new databases on tRFs, more differentially expressed tsRNAs associated with intimal hyperplasia have been identified. The purpose of this study is to explore the role and mechanism of these differentially expressed tsRNAs in neointimal hyperplasia. It will provide a new perspective for understanding the mechanism of vascular remodeling and may explore new biological markers or therapeutic targets for the treatment of cardiovascular diseases.

## Materials and methods

### Animal model

Animal experiments were performed in accordance with the National Institutes of Health Guide for the Care and Use of Laboratory Animals (2011) and approved by the Animal Ethics Committee of Southwest Medical University (No. 2020117). Experimental animals were euthanized according to the AVMA Guidelines for the Euthanasia of Laboratory Animals (2020).

Eight-week-old healthy male Sprague–Dawley (SD) rats were purchased from the Experimental Animal Center of Southwest Medical University and maintained in an environment with a temperature of 22 ± 2 °C and humidity of 55 ± 5%, with a 12-h light/12-h dark cycle of an artificial light source, with water and food available ad libitum. After 1 week of environmental acclimatization, rats were anesthetized by inhalation of 2% isoflurane, and the left and right external carotid arteries were isolated by an anterior median cervical incision. The distal end of the left external carotid artery was ligated, and the proximal end was clamped with an arterial clip. A small opening was made at the distal end of the left external carotid artery using ophthalmic scissors, through which a 2F balloon catheter (Edwards Lifesciences, Irvine, CA, USA) was inserted and advanced 1 cm into the common carotid artery. The balloon was inflated with 0.2 mL saline and the endothelial layer of the blood vessel was disrupted by pulling and twisting. This procedure was repeated three times. The control side of the carotid artery was dissected, but no invasive procedures were performed (balloon injury). After surgery the external carotid artery was ligated, the skin incision was closed, and penicillin was applied locally to prevent infection. After surgery, the rats were ventilated with pure oxygen for 5–10 min to facilitate recovery.

### Histologic examination

**Fig. 1:**
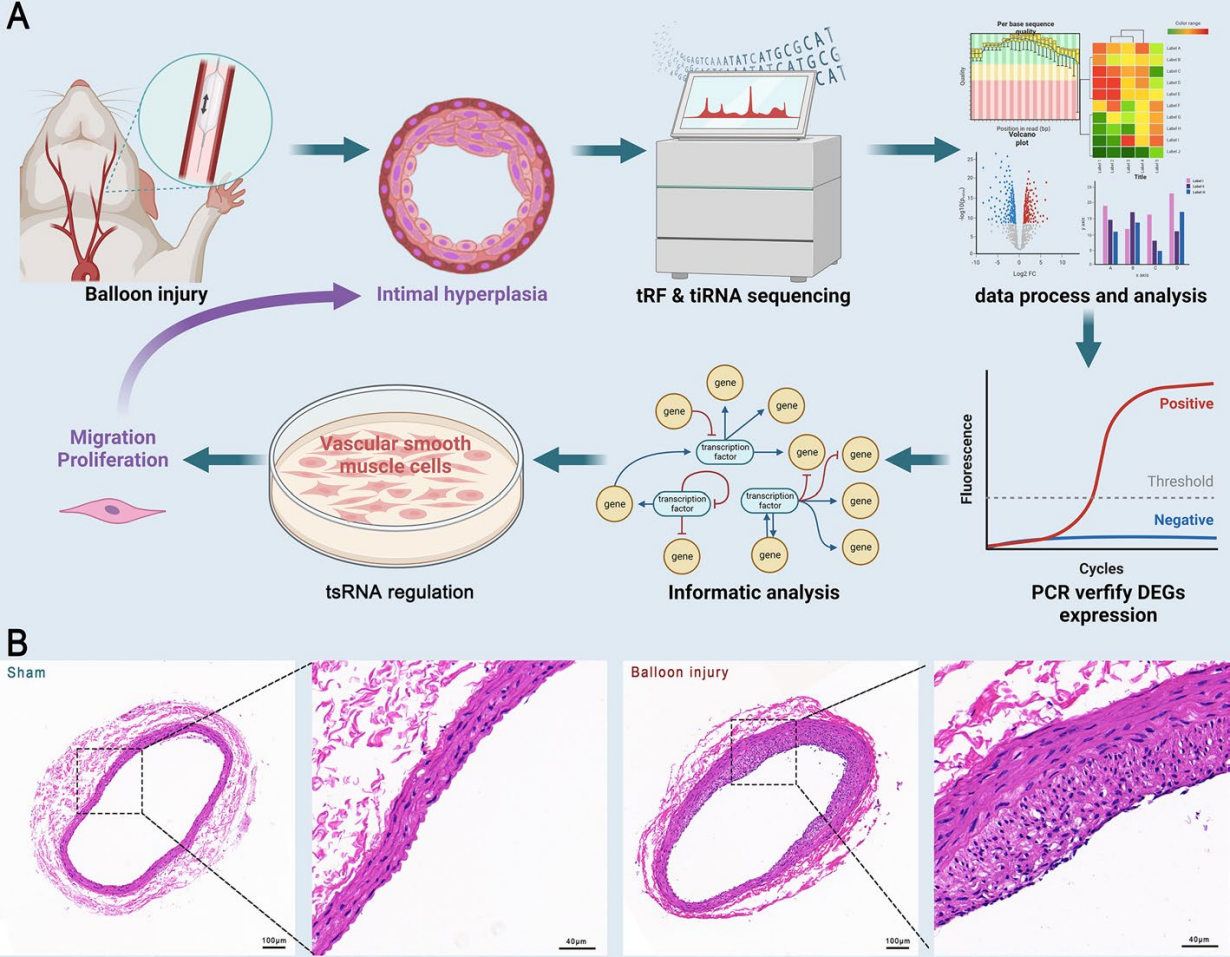
Experimental design and rat carotid artery neointimal formation. **A** Experimental procedure. First, balloon injury was used in the rat carotid artery to replicate the intimal hyperplasia model. Second, the carotid artery was subjected to small RNA sequencing to identify the differentially expressed tsRNAs (DE-tsRNAs). The expression of partial DE-tsRNAs was verified by qPCR. Then, the potential target mRNAs of the DE-tsRNAs were predicted by bioinformatic analysis. Finally, the expression of the DE-tsRNAs was modulated in vitro and in vivo to determine the function. **B** Intimal injury of the rat carotid artery was induced by a 2.0F Fogarty catheter. 14 days after surgery, the intima of the carotid artery was thickened and the lumen was narrowed. *n* = 5. Five sections per sample were measured for neointimal hyperplasia calculations

Two weeks after surgery, rats were euthanized with an overdose of pentobarbital (100 mg/kg). The control and experimental carotid arteries were isolated and removed, and the blood clot in the lumen of the vessels was washed out with saline. The blood vessels were cut into approximately 3 mm long vessel segments and then fixed in 4% paraformaldehyde for 1 h at room temperature. The blood vessels were embedded in paraffin, cut into 5-μm-thick sections, and stained with hematoxylin–eosin (0.2% hematoxylin, 1% eosin). The cross-section of the vessel was examined microscopically, the thicknesses of the intima and media were measured, and the intima/media area ratio (I/M) was calculated (Fig. 1A).

Immunohistochemical staining for fibromodulin (FMOD) was performed on paraffin sections of rat carotid arteries. First, paraffin sections of rat carotid arteries were subjected to antigen retrieval and non-specific antigen blocking. The sections were then incubated with FMOD antibody (Abcam # ab267465, Cambridge, UK). Finally, biotinylated secondary antibodies were used for color development.

### Small RNA sequencing and data analysis

Small RNA sequencing was performed by Aksomics Biotechnologies LLC (Shanghai, China). Rats were euthanized 14 days after surgery, and the control and the experimental carotid arteries were removed. Total RNA from the common carotid arteries was extracted with TRIzol reagent (Invitrogen, MA, USA. Catalog#: 15596026), and the integrity of the samples was checked by agarose gel electrophoresis and NanoDrop 2000 spectrophotometer (Thermo Fisher Scientific, MA, USA). RNA modifications of tRNA were removed for efficient reverse transcription using the rtStar™ tRF&tiRNA Pretreatment Kit (Catalog# AS-FS -005, Arraystar, MD, USA) [17]. Total RNA from the sample was then ligated to 3′ and 5′ adaptors, and cDNA was synthesized and amplified using RT primers and amplification primers (Illumina, CA, USA). PCR products of approximately 134–160 bp were extracted from the PAGE gel, purified and quantified using a 2100 Bioanalyzer (Agilent, CA, USA). The cDNA libraries were diluted and then sequenced on a NextSeq 500 system (Illumina, CA, USA), and data were collected.

Before data analysis, sequencing reads were removed of 5′ and 3′ adaptors and aligned using Bowtie software (Johns Hopkins University). Only 1 mismatch with mature or precursor tRNA sequences was allowed. Statistical analysis of the alignment (mapping ratio, read length, fragment sequence bias) was used to determine whether the results could be used for subsequent data analysis. If so, expression profiles and differentially expressed tsRNA were calculated. For confirmation, the sequencing results were compared with the tRF/tiRNA sequences in the database. A total of five databases were used in this study, namely GEO (https://www.ncbi.nlm.nih.gov/geo/), GtRNAdb (http://gtrnadb.ucsc.edu/index.html), tRFdb (http://genome.bioch.virginia.edu/trfdb/), MINIbase (http://cm.jefferson.edu/MINTbase/), and miRBase (http://www.mirbase.org/index.shtml). We have screened differentially expressed tRFs for carotid artery remodeling in previous work [17], and the databases used at that time were GEO, GtRNAdb, tRFdb, and tRF2Cancer (https://rna.sysu.edu.cn/tRFfinder/index.php). In this experiment, a new database (MINIbase, which includes nuclear and mitochondrial tRNA fragments) was used, and the data from the previously used databases (GEO, GtRNAdb, and tRFdb) were also updated. Therefore, there are differences in the differentially expressed tRFs profiles between the control and experimental groups that were screened out in the two experiments. In presenting the results of the second experiment, we omit the same results as in the first experiment and present the newly discovered differentially expressed tRFs. Finally, principal component analysis (PCA) and correlation analysis of tsRNA expression were calculated in the R environment, and pie charts, Venn plots, hierarchical clustering, scatter plots, and volcano plots were generated.

### Reverse transcription-polymerase chain reaction (RT-PCR)

Total RNA from common carotid arteries was extracted with TRIzol reagent according to the manufacturer’s instructions and assayed for concentration and purity using the NanoDrop 1000. RNA samples were pretreated with the rtStar™ tRF&tiRNA Pretreatment Kit to remove RNA modifications and ligate 3′ and 5′ adaptors. Samples were hybridized with reverse transcription primers and ligated with 5′-adaptors using the rtStar™ First-Strand cDNA Synthesis Kit (Catalog# AS-FS -003, Arraystar). Reverse transcription protocols were then performed on a thermal cycler (catalog# 1861096. Bio-Rad, CA, USA). The cDNA samples were hybridized with PCR-specific primers using the 2X PCR Master Mix (catalog# AS-MR-006-5. Arraystar) and amplification protocols were performed on the QuantStudio 5 Real-Time PCR System (Applied Biosystems, MA, USA).

### Bioinformatics analysis

The potential mRNA targets of tRF & tiRNA were predicted from the sequences using miRanda (https://mirdb.org) and TargetScan (www.targetscan.org). The biological process, cellular component and molecular function of the predicted target genes were analyzed using the Gene Ontology project (http://www.geneontology.org). Gene pathway analysis was performed using the KEGG PATHWAY database (www.genome.ad.jp/kegg/). Genes associated with intimal hyperplasia were downloaded from GeneCards (www.genecards.org) and compared with the predicted genes. The overlapping genes were divided into genes promoting intimal hyperplasia and genes inhibiting intimal hyperplasia, and genes promoting intimal hyperplasia were selected for further analysis and experiments.

### Proteomic detection and analysis

Proteomic detection of rat carotid arteries was performed by Aksomics (Shanghai, China). Rat carotid arteries were lysed with RIPA lysate and protease inhibitors, and protein concentration was measured. Peptide samples were obtained by acetone precipitation, protein redissolution, reduction, alkylation, and trypsin digestion. Peptide samples were desalted and dried under vacuum. Peptides were redissolved to 1 µg/µl in buffer. 2 µg of peptide from each sample was separated on an EASY-nLC1200 nano-UPLC liquid phase system (Thermo Fisher Scientific), detected by a Q-EXactive mass spectrometer (Thermo Fisher Scientific), and analyzed on a 100 μm ID × 15 cm reversed-phase chromatography column (Reprosil-Pur 120 C18-AQ, 1.9 μm, Dr. Math). The sample flow rate through the chromatography column was 300 nL/min and the gradient time was 120 min.

The acquired raw files were processed using MaxQuant (1.5.6.0). The protein database was obtained from the UNIPROT database (Uniprot_rat_2018_10). The type of quantification was label-free quantification (LFQ). Three groups of biological replicates were set up in the experiment, and Student’s t-test was performed. Proteins with expression fold change (ratio A/B) > 1.25, *P* value < 0.05 were defined as significantly different.

### Cell culture and passaging

Rat thoracic aortic smooth cells were cultured from SD rat thoracic aorta. Eight-week-old healthy male SD rats were euthanized with an overdose of pentobarbital (100 mg/kg). The skin of the anterior chest wall of the rats was disinfected with alcohol, and the anterior median incision was made on the chest wall to separate and remove the thoracic aorta. The removed thoracic aorta was soaked in a balanced salt solution containing penicillin and streptomycin, and the adventitia was stripped. The thoracic aorta was cut longitudinally and placed in 1% type I collagenase intimal side down and incubated at 37 °C for half an hour. The medial wall of the thoracic aorta was carefully rinsed with DMEM medium containing 5% fetal bovine serum to elute vascular endothelial cells. The thoracic aorta, with the adventitia and intima removed, was cut into small pieces with ophthalmic scissors and spread evenly in DMEM medium containing 10% fetal bovine serum. After 4–7 days, the cells had crawled away from the tissue blocks. Tissue pieces were removed. Adherent cells were digested with 0.1% trypsin and smooth muscle actin was detected by immunofluorescence (smooth muscle alpha-actin antibody was purchased from Abcam. Catalog # AB5694). When the alpha-actin-positive cells were > 95%, the cells were passaged and the 4th to 7th passages were used for experiments.

### Small RNA transfection

Synonymous nucleotide sequences (analog) of tsRNAs were synthesized by RiboBio (Guangzhou, China), scrambled small RNAs were used as a control. 2 × 10^5^ VSMCs were seeded in six-well plates. When the cells were attached to the wall and reached approximately 70% confluence, 50 μmol/L tsRNAs analog was transfected into the cells using Ribo FECT CP Transfection Reagent (Catalog # C10511) according to the manufacturer’s instructions. After 48 h, the cells were used for the following experiments.

### Dual-luciferase reporter assay

The Gp Mirglo dual luciferase vector (GenePharma, Shanghai, China) with SacI and XhoI restriction sites was digested with the appropriate enzymes. The sequence of wild-type FMOD 3'UTR (AAATCCACAAAAGCCAAACCAGCTTGTTTGAACCAGGGAGTGCCACATGTGGAGCAAGGCTGCCCTGCC) or its mutated sequence (AAATCCACAAAAGCCATTGGTGCTTGTTTGATGGTCCCAGTGCCACATGTGGAGCAAGGCTGCCCTGCC) was cloned into the vector. The vector plasmids were amplified in the competent cells and the cloned sequences were identified by RNA sequencing. The verified plasmids were then extracted in large quantities. HEK293T cells were cultured in a 6-well plate and grown to 60–70% confluence. Wild-type or mutated FMOD sequence plasmids, positive control (PC) plasmid, empty vector, tRF-Glu-CTC analog or negative control (NC) oligonucleotide were transfected into HEK293 cells for 24 h. Relative firefly / renilla luciferase activity was detected using the dual-luciferase reporter assay system (Promega, USA) on a Synergy HTX multimode reader (BioTek, USA).

### Enzyme-linked immunosorbent assay (ELISA)

In vitro cultured cells were lysed with RIPA buffer and protein concentration was determined. According to the manufacturer's instructions, the levels of FMOD and HSP20 were determined using the quantitative ELISA kits (FANKEL IndustrialCo., Ltd, Shanghai, China. CAT No. F21350 for FMOD and CAT No. F3334 for HSP20).

### Inhibition of tRF-Glu-CTC expression in rat carotid artery

After balloon injury of the carotid artery in rats, chemically modified stable tRF-Glu-CTC antisense, which is fully complementary to tRF-Glu-CTC, was injected around the artery, and scrambled small RNA was used as a control. To increase the stability of the RNA and its ability to bind to mammalian cells, chemical modifications were made to the RNA molecules (Stable ™ siRNA, two ends of the RNA sequence were modified with a thio backbone, the 3′ end was modified with cholesterol, and the entire chain was modified with methoxy). The sequences were synthesized by GenePharma (Shanghai, China), and the RNAs were subjected to HPLC purification and MALDI-TOF analysis. RNA purity must exceed 97% before use in subsequent experiments. Chemically modified antisense was dissolved in RNase-free sterile water at a concentration of 10 μg/μL. 0.5 μL siRNA (5 μg) was added to 0.45 μL RNase-free sterile water followed by 0.55 μL Lipofectamine (24 μg). The mixture was incubated at room temperature for 30 min to form the siRNA-lipofectamine complex. The prepared solution was injected with a syringe at multiple points around the carotid artery on the operated side, and then repeated every 3 days [18]. On day 14 after surgery, the rats were euthanized, and the carotid arteries were excised for histological and molecular biological detection.

### Western blot

Rat carotid arteries were pretreated with RIPA lysis buffer and protease inhibitors (Beyotime Biotechnology, Shanghai, China). Total proteins were determined using a NanoDrop 1000 spectrophotometer (Thermo Fisher Scientific), separated by 10% SDS-PAGE gel electrophoresis, and transferred to PVDF membranes. The PVDF membranes were blocked with BSA (Sigma-Aldrich) and then hybridized with antibodies. Anti-FMOD antibody (catalog # ab267465) was purchased from Abcam (Cambridge, UK) and diluted 1:1000. Anti-Smad3 antibody (catalog # #9513) was purchased from Cell Signaling Technology (MA, USA) and diluted 1:1000. GAPDH was used as an internal reference and anti-GAPDH antibody was purchased from Abcam (catalog # ab9485) and diluted 1:1000. The PVDF membranes were incubated with a developing solution and developed in the Molecular Imager Gel Doc XR system (Bio-Rad Laboratories, Inc.). Western blots were analyzed with ImageJ software (National Institutes of Health).

### EdU cell proliferation assay

Cell proliferation was determined by the 5-ethynyl-2′-deoxyuridine (EdU) incorporation assay. Briefly, 1 × 10^4^ VSMCs were seeded in 96-well plates and grown to 50% confluence. EdU reagent (RiboBio Co. Ltd., Guangzhou, China) was incubated with the cells for 24 h, and then the fluorescent display solution was incubated with the cells for 2 h at room temperature. Finally, the cells were fixed with 4% paraformaldehyde for 30 min. Proliferating VSMCs were observed and counted under a fluorescence microscope, and the number of DAPI (4′,6-diamidino-2-phenylindole)-stained nuclei was used as an internal reference.

### Wound healing assay

A wound healing assay was used to evaluate the migration of VSMCs. 1 × 10^6^ VSMCs were cultured in a six-well plate at 37 °C for 24 h to 60–70% confluence. A cross-shaped wound was made smoothly with a pipette tip perpendicular to the monolayer surface of the VSMCs, and the wound width was measured under a light microscope. The wound width was photographed under a microscope after 24 h to calculate the wound healing rate.

### Transwell migration assay

The transwell assay was also used to detect the migration of VSMCs. 1 × 10^5^ VSMCs were seeded with serum-free culture medium on the 6.5 mm transwell inserts with 8.0 µm porous polycarbonate membrane (catalog # 3422, Corning, Inc., MA, USA). The inserts were then placed in a 6-well plate with a culture medium containing 10% fetal bovine serum for 24 h to allow VSMCs to migrate through the micropores from the inside to the outside of the inserts. Finally, the VSMCs were fixed with 4% paraformaldehyde and stained with hematoxylin. Non-migrated VSMCs within the inserts were wiped off with a cotton swab. The migrated VSMCs on the outside of the inserts were observed and counted under a light microscope.

### Statistical analysis

Each experiment was repeated at least three times (*n* ≥ 3). Results are expressed as the mean ± standard error of the mean (SEM). Data analysis and statistical processing were performed using Prism 9.0 software (GraphPad, Inc.). A two-tailed, unpaired Student’s t-test was used for comparisons between the two groups. A* P* value < 0.05 was considered statistically significant.

## Results

### Neointima formation induced by balloon injury of the rat carotid artery

Fourteen days after balloon injury of rat carotid arteries, rats were euthanized, and the control and the experimental carotid arteries were removed for histological sectioning. Neointimal formation was observed in the balloon-injured carotid artery compared with the sham-operated control (Fig. 1B).

### Small RNA sequencing revealed the differentially expressed tsRNA expression profiles

**Fig. 2:**
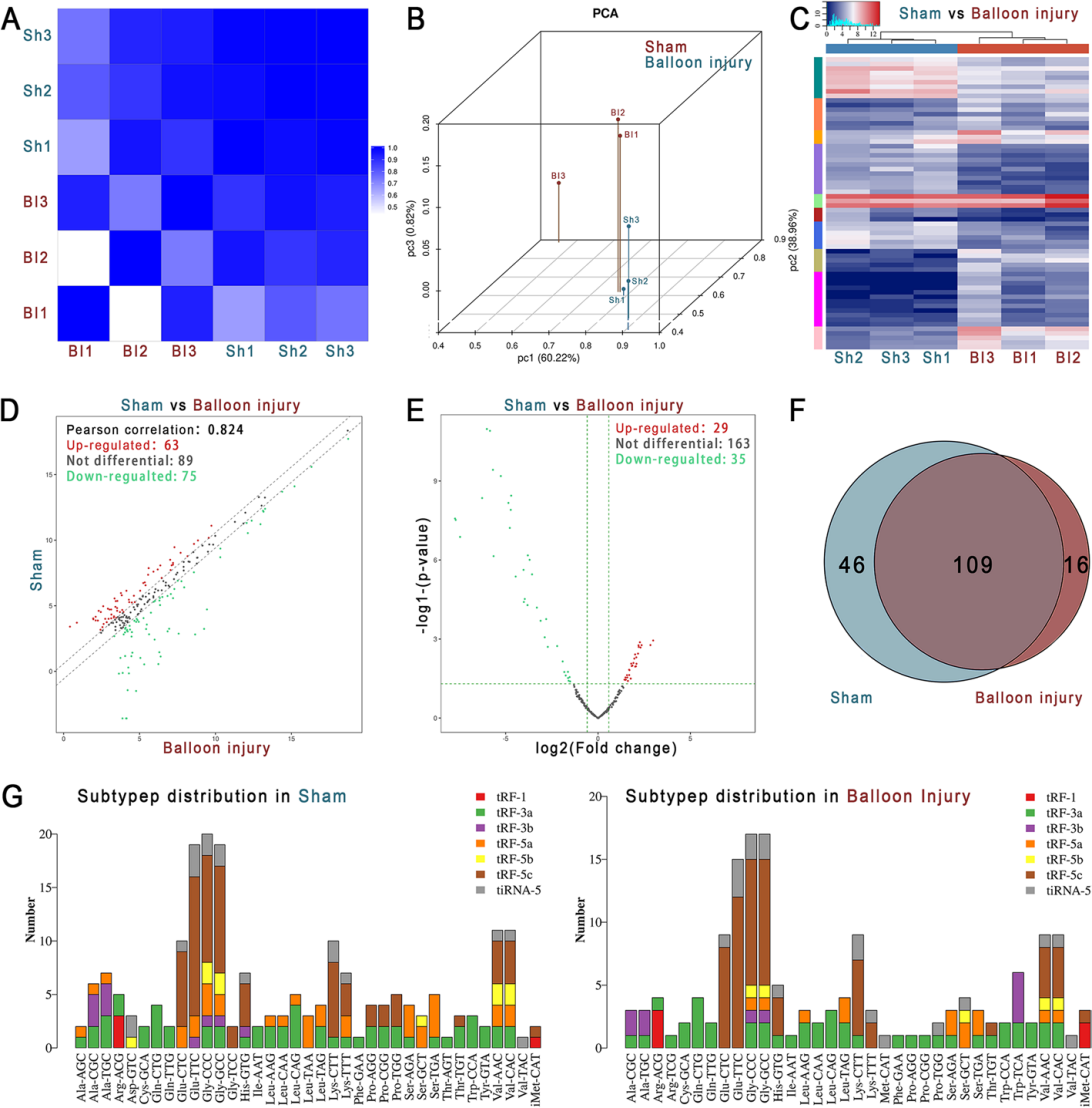
Small RNA sequencing.** A** Heatmap of the correlation coefficient of the carotid artery samples. Blue represents the samples with a high correlation coefficient, and white represents the samples with no similarity. **B** Primary component analysis. The location of the colored dots shows the main character of the sample. The spatial distance represents the similarity of the samples. **C** The hierarchical clustering heatmap for tsRNAs. The color in the panel represents the relative expression level. Blue represents an expression level below the mean and red represents an expression level above the mean. **D** The scatter plot between the sham group and the balloon injury group for tsRNAs. Red dots (upregulated) and green dots (downregulated) indicate ≥ 1.5 fold change between the two groups. Gray dots indicate non-differentially expressed genes. **E** The volcano plot of tsRNAs. Red (up-regulated) and green (down-regulated) dots indicate statistically significant differentially expressed tRFs & tiRNAs with fold change ≥ 1.5 and *P*-value ≤ 0.05. **F** Venn diagram showing the number of tsRNAs expressed in the sham group and balloon injury group. **G** The subtypes of tsRNAs. The X-axis represents the origin of tRNAs and the Y-axis shows the number of tsRNAs. *n* = 3. *Sh* sham, *BI* balloon injury

Small RNA sequencing was performed on carotid arteries and 14-40nt small RNAs were compared with information in tRNA databases to screen for tRF & tiRNA. Compared with the healthy carotid arteries, the expression profiles of tsRNAs in carotid arteries with intimal hyperplasia were significantly different (Fig. 2A–G).

**Fig. 3:**
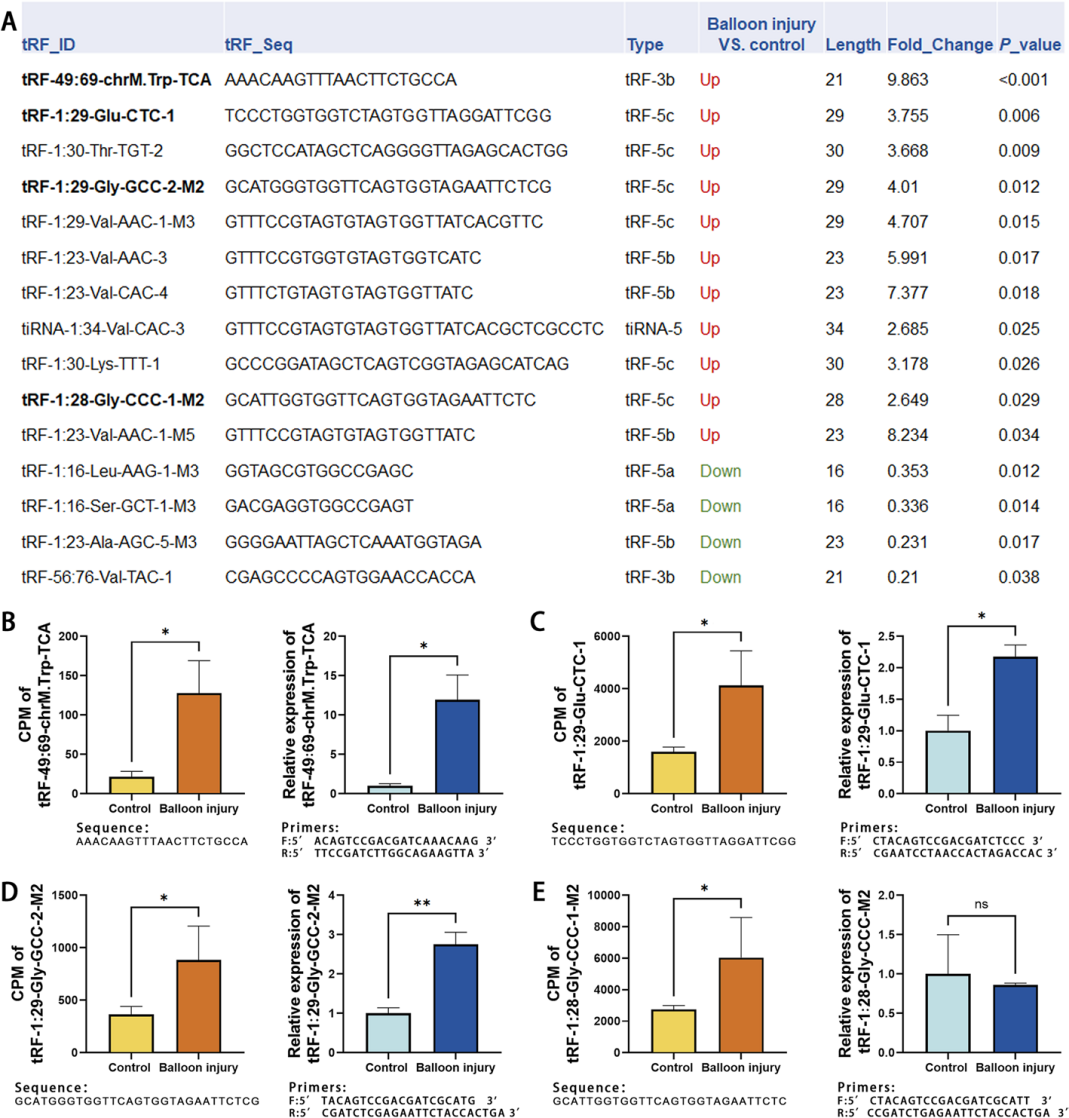
Differentially expressed tsRNAs in the hyperplastic intima of rat carotid artery. **A** The abundance of tsRNAs and miRNAs was evaluated by their sequencing counts and were normalized as counts per million of total aligned reads (CPM). The top 15 up- and down-regulated tsRNAs in hyperplasic intima were listed based on their statistical significance, CPM, and expression change (fold change). **B–E** The expression differences of tRF-49:69-chrM.Trp-TCA, tRF-1:29-Glu-CTC-1, tRF-1:29-Gly-GCC-2-M2 and tRF-1:28-Gly-CCC-M2 in the sham surgery group and the balloon injury group were verified by qPCR. Their expression was all upregulated in the balloon injury group except for tRF-1:29-Gly-GCC-2-M2 by qPCR. *n* = 3, data are expressed as mean ± standard error, *P < 0.05, **P < 0.01, ns indicates for not statistically significant

Some tsRNAs were significantly upregulated in carotid arteries with intimal hyperplasia (Fig. 3A). Selected according to the statistical *P* value and CPM (reads per million mapped) of the expression data, some of the tsRNAs were tested by RT-PCR to verify their expression in normal and injured rat carotid arteries (Fig. 3B–E). tRF-49:69-chrM.Trp-TCA, tRF-1:29-Glu-CTC-1, and tRF-1:29-Gly-GCC-2-M2 were all highly expressed in balloon-injured carotid arteries and were consistent with the small RNA sequencing results.

### Bioinformatic analysis of DE-tsRNAs and verification of expression of putative target genes

**Fig. 4:**
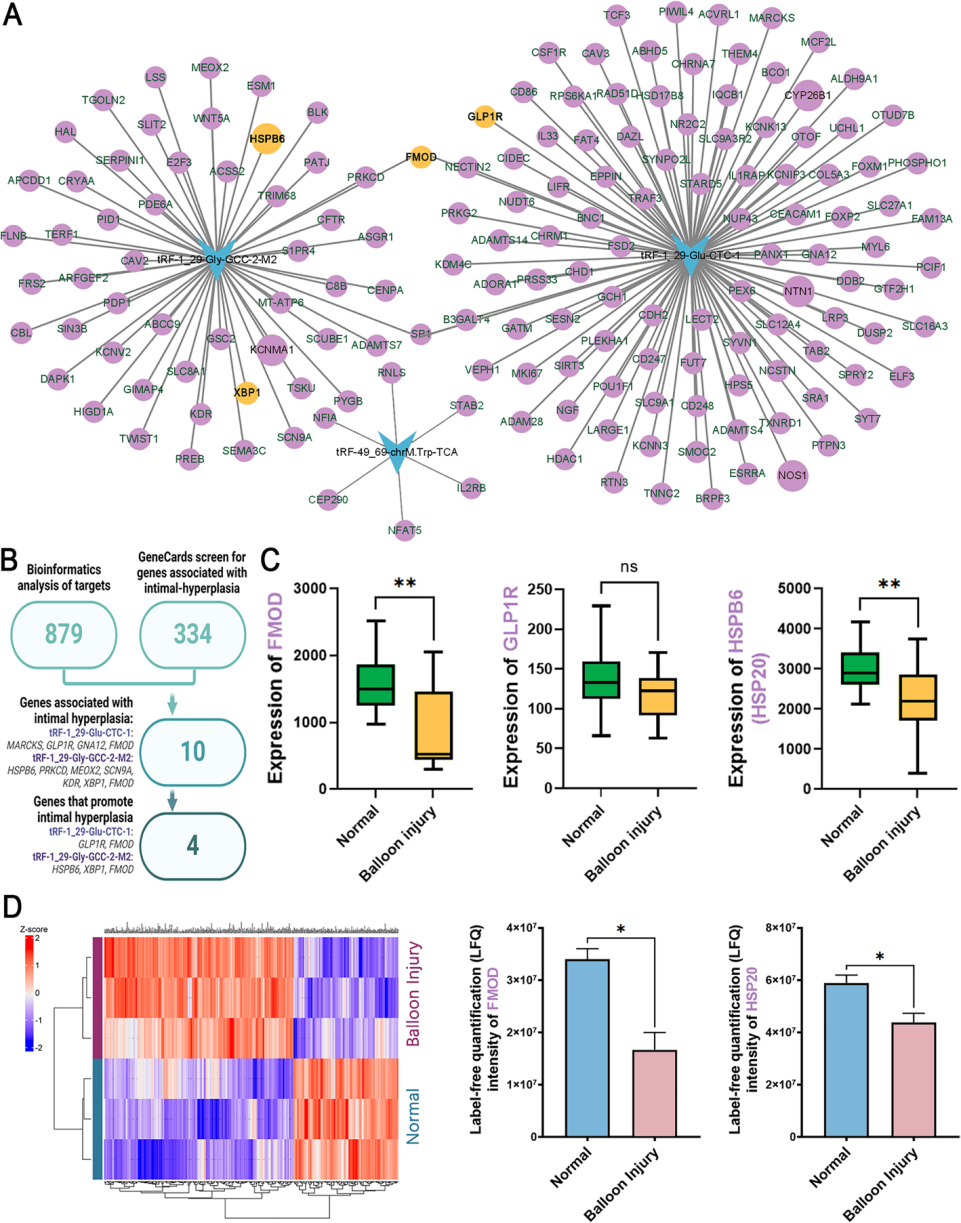
Bioinformatic analysis of differentially expressed tsRNAs (DE-tsRNAs) and target gene screening. **A** Potential target genes of DE-tsRNAs were predicted by miRanda and TargetScan. The clustering map of DE-tsRNAs and target genes was generated by Cytoscape. **B** The process of screening for target genes that may be involved in intimal hyperplasia. First, the GeneCard database was used to select genes associated with intimal hyperplasia from the predicted list of potential target genes. Then, a GEO dataset GSE164050 (*n* = 4) was used to screen the genes downregulated in the balloon injury rat model, and 4 genes GLP1R, FMOD, HSPB6 (HSP20), and XBP1 were focused. **C** According to GSE164050, the mRNA expression of FMOD and HSP20 was decreased in the balloon injury, the expression of XBP1 was undetectable, and the expression change of GLP1R was not statistically significant. **D** Carotid proteomic detection confirmed that the protein levels of FMOD and HSP20 were decreased in the balloon injury group. *n* = 3, data are expressed as mean ± SD. *P < 0.05, **P < 0.01; ns indicates not statistically significant

Based on the sequences of tRF-49:69-chrM.Trp-TCA, tRF-1:29-Glu-CTC-1, and tRF-1:29-Gly-GCC-2-M2, TargetScan and miRanda predicted that they may have 879 target genes (Fig. 4A). The 334 genes related to intimal hyperplasia were downloaded from GeneCards (https://www.genecards.org) and matched with the predicted target genes. Among them, 10 genes are predicted target genes of tsRNAs. They are tRF-1_29-Glu-CTC-1’s potential target genes MARCKS, GLP1R, GNA12, FMOD, and tRF-1_29-Gly-GCC-2-M2 potential target genes HSPB6 (HSP20), PRKCD, MEOX2, SCN9A, KDR, XBP1, FMOD (Fig. 4B). Among these genes, GLP1R [19, 20], FMOD [21], HSP20 [22], and XBP1 [23] have been reported to influence vascular intimal hyperplasia.

Data set GSE126627 of the rat carotid artery balloon injury model was downloaded from GEO (Gene Expression Omnibus, NIH. https://www.ncbi.nlm.nih.gov/geo/). Analysis showed that the expression of FMOD and HSPB20 was significantly reduced in the balloon-injured rat carotid artery. The expression of GLP1R was slightly reduced in the injured artery, but there was no statistical difference (Fig. 4C). XBP1 expression was not detected.

Proteomic detection of rat carotid arteries showed that protein levels of FMOD and HSP20 were reduced in carotid arteries with neointima formation after balloon injury compared with healthy rat common carotid arteries (Fig. 4D).

Taken together, both mRNA expression and protein levels of FMOD and HSP20 were decreased in the vessel wall with neointima formation.

### FMOD is the target gene of tRF-1:29-Glu-CTC-1

**Fig. 5:**
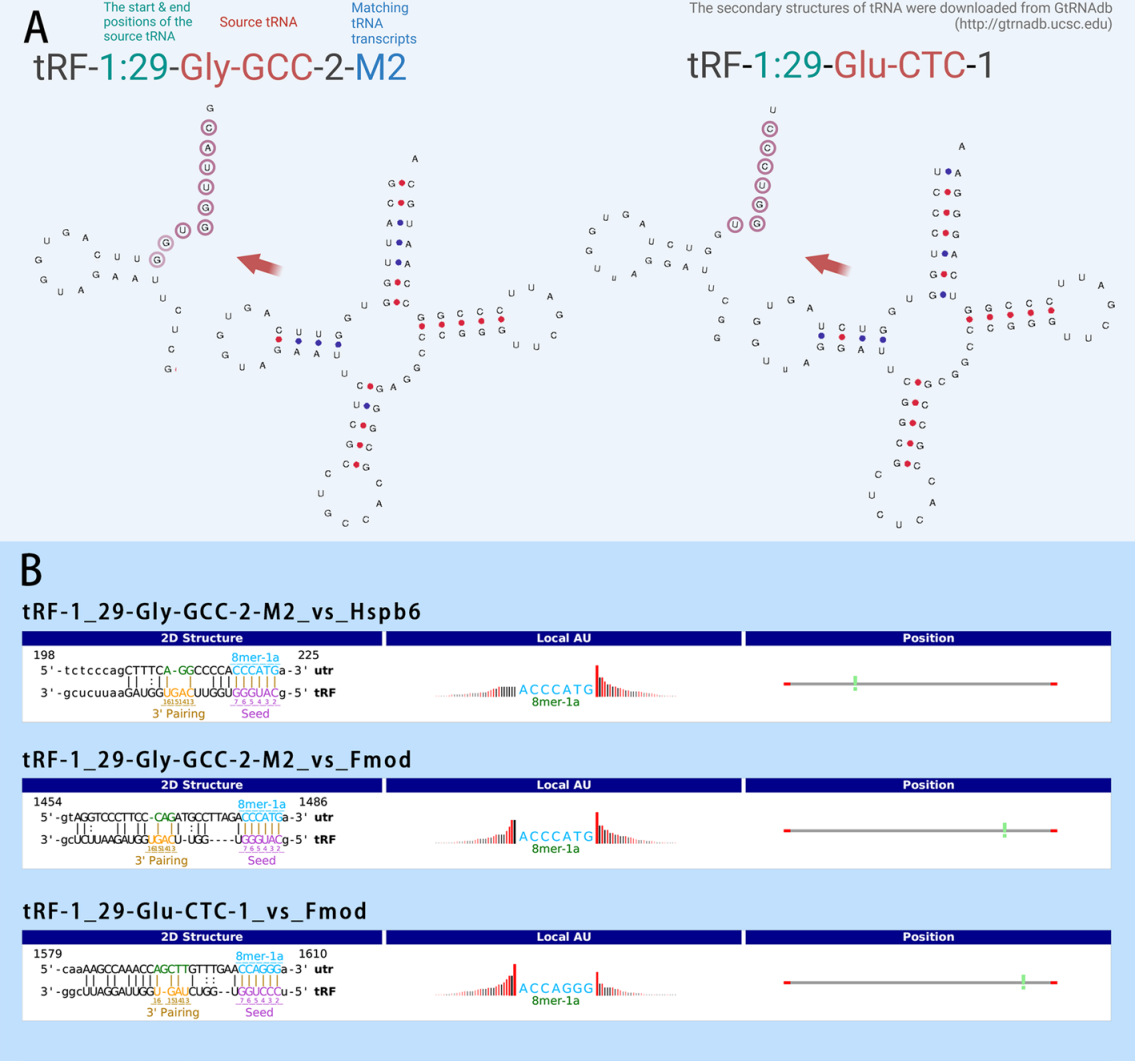
Structures of tRF-1:29-Gly-GCC-2-M2 and tRF-1:29-Glu-CTC-1 and the interactions with putative target genes. **A** tRF-1:29-Gly-GCC-2-M2 and tRF-1:29-Glu-CTC-1 are derived from the 5′ ends of tRNA-Gly-GCC and tRNA-Glu-CTC, respectively. In this schematic, bases putatively bound to target genes are highlighted with circles. **B** According to the sequence prediction of tRF-1:29-Gly-GCC-2-M2 and tRF-1:29-Glu-CTC-1, they may bind to the 3′ untranslated region (UTR) of HSP20 or FMOD

The tRNA sources of tRF-1:29-Gly-GCC-2-M2 and tRF-1:29-Glu-CTC-1 and their possible binding sites to their putative target genes HSP20 (HSPB6) and FMOD are shown in Fig. 5A and B. Among them, tRF-Gly-GCC may bind to the mRNA of HSP20 or HSP20, and tRF-Glu-CTC may bind to the mRNA of FMOD.

**Fig. 6:**
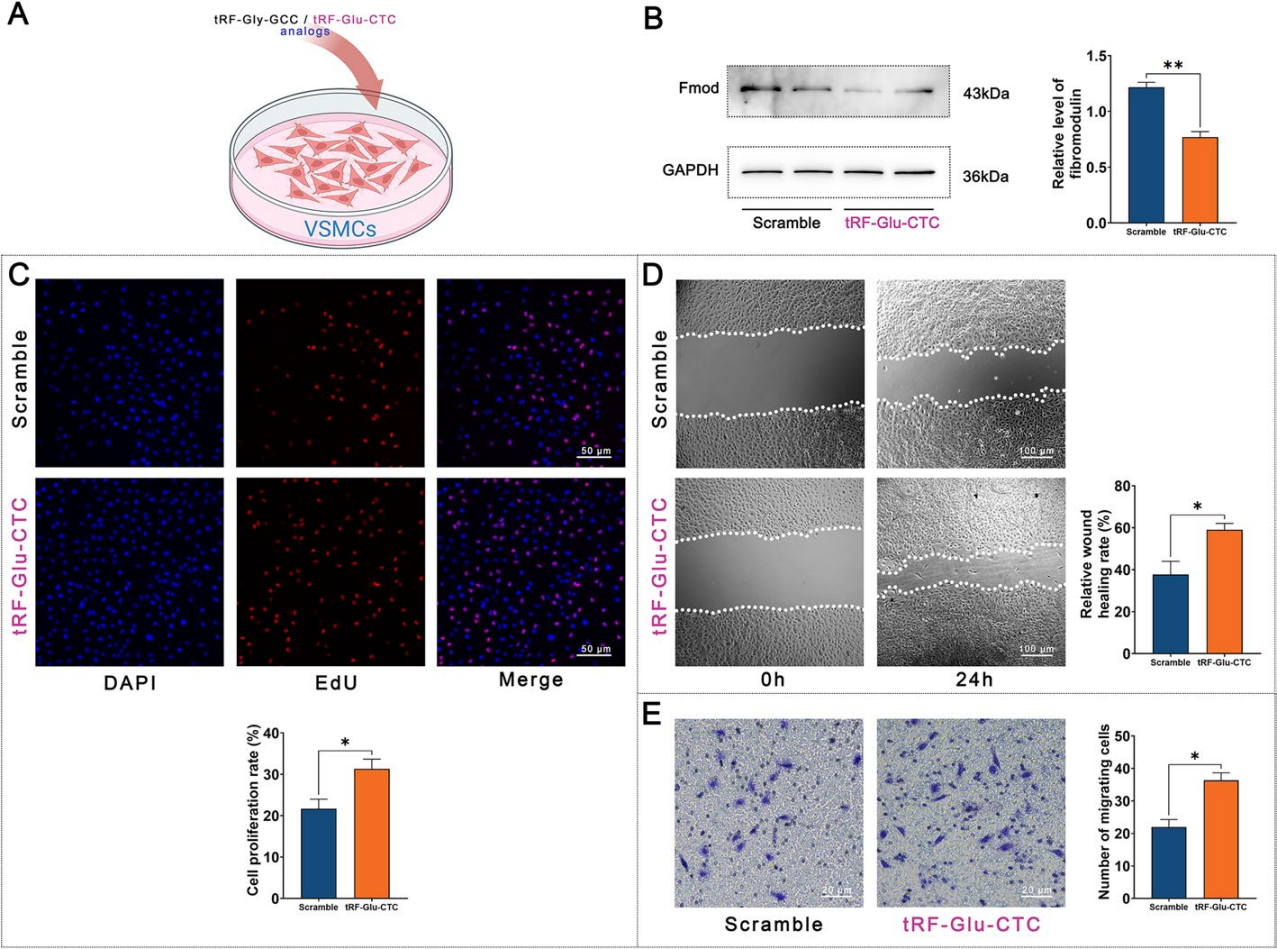
tRF-Glu-CTC inhibited fibromodulin (FMOD) levels in vascular smooth muscle cells (VSMCs) and upregulated VSMC proliferation and migration. **A** According to bioinformatics analysis, tRF-Glu-CTC may be a negative regulator of fibromodulin (FMOD) in VSMCs. Therefore, polynucleotide analogs of tRF-Glu-CTC were synthesized and transfected into rat thoracic aortic VSMCs by liposomes. **B** Compared with the transfection of scrambled RNA sequences, the transfection of tRF-Glu-CTC analog inhibited the level of FMOD in VSMCs. **C** Transfection of tRF-Glu-CTC increased the proliferation of VSMCs in the EdU incorporation assay. **D** Transfection of tRF-Glu-CTC increased the migration of VSMCs in the wound healing assay. **E** Overexpression of tRF-Glu-CTC also increased the migration of VSMCs in the wound healing assay. *n* = 5, data are expressed as mean ± standard error, *P < 0.05, **P < 0.01

The analogs of tRF-Gly-GCC and tRF-Glu-CTC were each transfected into rat aortic smooth muscle cells (VSMCs), and then the protein levels of FMOD and HSP20 were detected in the cell lysate (Fig. 6A). ELISA showed that tRF-Glu-CTC decreased the FMOD levels in VSMCs, but tRF-Gly-GCC had no significant effect on the FMOD and HSP20 levels in VSMCs (Additional file 1: Fig. S1). Western blot confirmed the decrease in FMOD level after tRF-Glu-CTC transfection (Fig. 6B). Furthermore, proliferation (Fig. 6C) and migration (Fig. 6D and E) of VSMCs were increased after the transfection of tRF-Glu-CTC.

**Fig. 7:**
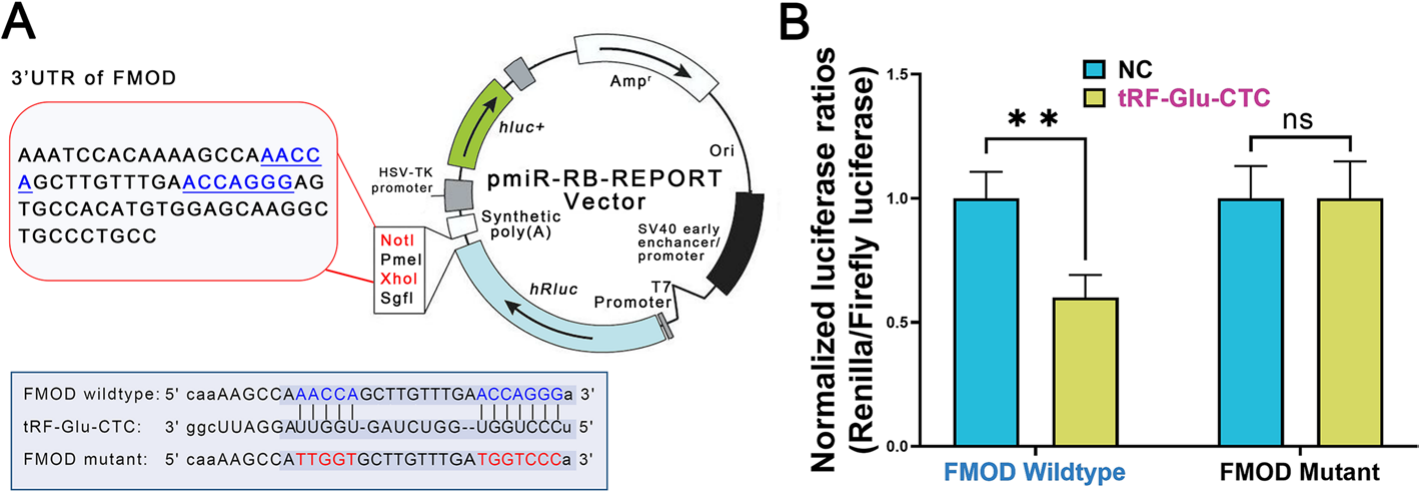
tRF-Glu-CTC affected the transcription of FMOD.** A** The 3′UTR of FMOD, which is predicted to interact with tRF-Glu-CTC, was ligated into the pmiR-RB-Reporter vector. **B** Dual-luciferase reporter assay performed in the HEK 293 cell line showed that the analogs of tRF-Glu-CTC reduced the relative luciferase activity of the expression vector constructed from wild-type FMOD mRNA and the firefly luciferase gene but did not affect the relative luciferase activity of the expression vector constructed from the mutated FMOD mRNA sequence. *n* = 3, data are expressed as mean ± standard error, **P < 0.01, ns indicates for not statistically significant

A dual luciferase reporter gene assay was used to determine the binding of tRF-Glu-CTC to FMOD mRNA. If tRF-Glu-CTC can bind to the specific loci of FMOD mRNA, it will affect the transcription of the Firefly luciferase reporter gene downstream of the vector and reduce the relative Firefly/Renilla luciferase activity. If tRF-Glu-CTC is unable to bind to FMOD mRNA, it will not affect the transcription of the downstream Firefly luciferase reporter gene and the relative Firefly/Renilla luciferase activity will remain unchanged. The wild-type FMOD mRNA fragment complementary to the tRF-Glu-CTC sequence or the FMOD mRNA fragment mutated in the complementary sequence was ligated into the pmiR-RB-REPORT vector (Fig. 7A). The vector and tRF-Glu-CTC were then co-transfected into HEK293 cells. Experiments showed that co-transfection of wild-type FMOD mRNA and tRF-Glu-CTC analogs reduced the relative activity of the Firefly/Renilla luciferase of the luciferase reporter system, but co-transfection of mutant FMOD mRNA and tRF-Glu-CTC analogs had no effect on the relative activity of the Firefly/Renilla luciferase of the reporter system (Fig. 7B). It was shown that tRF-Glu-CTC can bind to FMOD mRNA as bioinformatically predicted.

### Reduction of tRF-Glu-CTC expression contributed to attenuation of balloon injury-induced neointimal hyperplasia

**Fig. 8:**
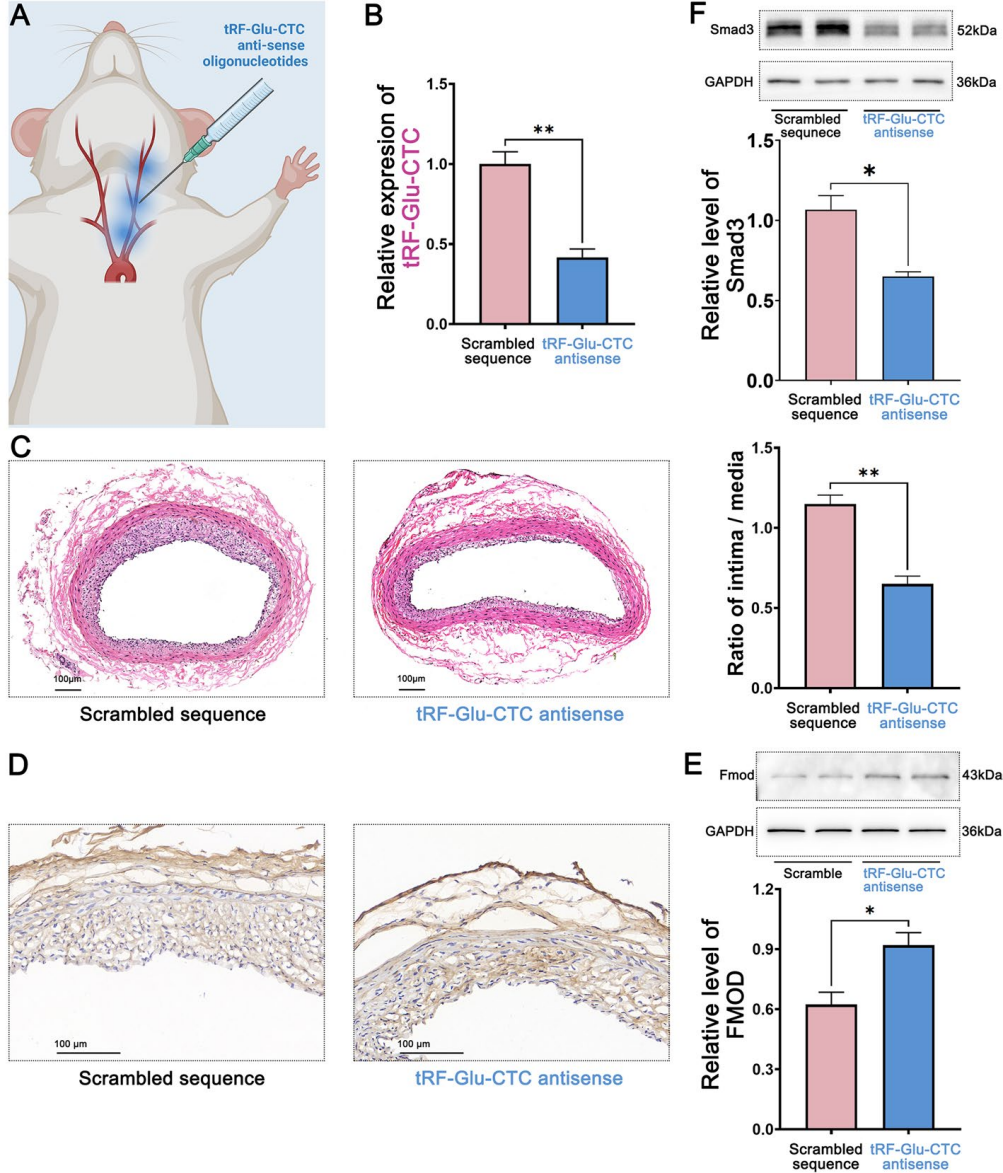
Transfection of tRF-Glu-CTC antisense attenuated neointimal formation after carotid artery balloon injury in rats. **A** Multipoint injection of chemically modified tRF-Glu-CTC antisense around the arteries on the side of surgery and repeated every 3 days until postoperative day 14 after carotid balloon injury in rats. **B** Detection of tRF-Glu-CTC expression in rat carotid arteries at 14 days. Compared with the control group injected with scrambled sequence, the expression of tRF-Glu-CTC in the carotid artery of the tRF-Glu-CTC antisense injection group decreased. **C** The cross section of the rat carotid artery showed that the area of carotid intimal hyperplasia was less in the tRF-Glu-CTC antisense treatment group than in the control group. The intima/media ratio was decreased in the tRF-Glu-CTC antisense treatment group. **D** Immunohistochemical staining for FMOD on rat carotid arteries showed that rats transfected with tRF-Glu-CTC antisense increased the positive staining of FMOD in the vessel wall after carotid artery intima injury compared with the control group transfected with scrambled RNA sequence. **E** Western blotting showed the increased level of FMOD in the rat carotid artery transfected with tRF-Glu-CTC antisense after balloon injury. **F** Transfection of tRF-Glu-CTC antisense decreased the level of Smad3, the major effector of TGF-β1. *n* = 5, data are expressed as mean ± standard error, *P < 0.05, **P < 0.01

After replicating the rat carotid artery intimal injury model, the chemically modified stabled tRF-Glu-CTC antisense was injected into the tissue surrounding the artery (Fig. 8A). Carotid arteries were excised 14 days after surgery for qRT-PCR detection. Compared to the control group injected with scrambled sequence, the expression of tRF-Glu-CTC decreased by 58.33% in the carotid arteries of rats injected with tRF-Glu-CTC antisense (Fig. 8B). Histological sections of the carotid arteries showed that the neointimal area was reduced in the carotid arteries of rats injected with tRF-Glu-CTC antisense, and the intima/media area ratio of the arteries was reduced by 58.48% (Fig. 8C). Immunohistochemical staining for FMOD on rat carotid artery sections showed that transfection of tRF-Glu-CTC antisense increased FMOD levels compared with transfection of scrambled RNA (Fig. 8D). Western blotting also confirmed the 32.25% increase in FMOD in the tRF-Glu-CTC antisense transfected carotid artery (Fig. 8E). The level of Smad3, an indicator of TGF-β1 activity [24, 25], was decreased (Fig. 8F).

## Discussion

tRNAs are conserved and abundant RNAs. In addition to their key role in protein translation, they are also an important source of small non-coding RNAs. Dicer or angiogenin (ANG) cleaves precursor tRNA or mature tRNA at the D-loop, T-loop or anticodon loop to form 14–30 nucleotides (nt) tRNA-derived small RNAs (tsRNAs) or 31–40 nt tRNA halves (tiRNAs) [11]. tsRNAs can affect reproduction, development, cancer, viral infection, and cardiovascular disease by regulating mRNA stability and protein translation [26–28].

In this study, small RNA sequencing and tsRNA library were used to screen differentially expressed tsRNAs in the carotid artery wall after vascular injury, and then focused on tRF-Glu-CTC by functional prediction and biological verification. In vitro, overexpression of tFR-Glu-CTC was found to negatively regulate fibromodulin (FMOD) levels and increase vascular smooth muscle cell proliferation and migration. FMOD is a transforming growth factor-β (TGF-β) modulator that antagonizes TGF-β activity [29–32]. TGF-β1 plays an important role in promoting intimal hyperplasia [33–35]. In vivo, TGF-β1 has been shown to be a potent stimulator of vascular smooth muscle cell (VSMC) proliferation [24, 25, 33, 36]. Mechanistically, TGF-β1 is upregulated after arterial injury and stimulates VSMC proliferation and migration [37, 38] and extracellular matrix protein production [39]. Overexpression of FMOD in vein grafts attenuates neointimal hyperplasia by inhibiting TGF-β1 activity [21]. In our experiments, local administration of tRF-Glu-CTC antisense inhibited the expression of tRF-Glu-CTC after balloon injury in rat carotid artery, upregulated the level of FMOD and downregulated the level of Smad3 in the arterial wall and reduced the neointimal area.

tsRNAs have been identified as biomarkers or potential therapeutic targets in many cardiovascular diseases, including rheumatic heart disease [40], cardiac hypertrophy [13], heart failure [16], atherosclerosis [41], and aortic dissection [42]. There is evidence that tsRNAs regulate the proliferation [43] and phenotypic switching [44] of VSMCs. In our previous work, we also found that tRF-Gln-CTG induced vascular intimal hyperplasia by inhibiting FAS cell surface death receptors [17]. This study demonstrates that tRF-Glu-CTC affects neointimal formation by regulating FMOD and provides new insights into the role of tsRNA in VSMC biology.

This study has several limitations. First, the specific upstream mechanism of increased tRF-Glu-CTC expression after vascular injury remains to be elucidated. Second, the dynamic changes of tRF-Glu-CTC and FMOD in each layer of the vascular wall after vascular intimal injury were not observed in this study. Finally, this study was based on animal experiments in rats, and it is unclear whether human vascular remodeling is affected by tRF-Glu-CTC.

## Conclusions

**Fig. 9:**
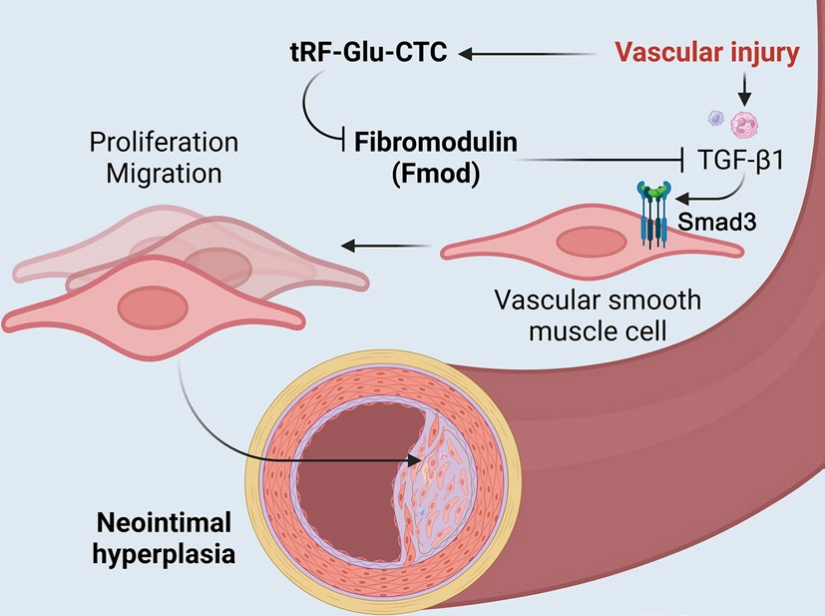
Mechanism of tRF-Glu-CTC promotion of neointimal hyperplasia. After vascular wall injury, TGF-β1 is produced by the recruitment of inflammatory cells (neutrophils/macrophages, etc.), which is a potent agonist of vascular smooth muscle cells and mediates neointimal formation by promoting proliferation and migration of VSMCs via Smad3. Vascular injury induced the expression of tRF-Glu-CTC, which suppressed fibromodulin (FMOD) levels through the RNA silencing mechanism. FMOD is a TGF-β1 antagonist that inhibits the activity of TGF-β1. Thus, tRF-Glu-CTC played a promoting role in the process of neointima formation by indirectly facilitating the activity of TGF-β1

After the vascular intimal injury, expression of the tRNA-derived small fragment tRF-Glu-CTC increases, thereby decreasing fibromodulin (FMOD) levels, which may inhibit neointima formation by antagonizing the activity of the TGF-β1/Smad3 signaling pathway (Fig. 9). Inhibition of tRF-Glu-CTC expression after intimal injury reduces neointimal area, which may be a promising approach to prevent vascular stenosis.

### Supplementary Information

**Additional file 1. Supplementary Figure 1.** ELISA showed that tRF-Glu-CTC inhibited fibromodulin (FMOD) levels in vascular smooth muscle cells (VSMCs). **A** According to bioinformatics analysis, tRF-Gly-GCC may be a negative regulator of fibromodulin (FMOD) and/or HSP20 (HSPB6) in VSMCs. Therefore, polynucleotide analogs of tRF-Gly-GCC were synthesized and transfected into rat thoracic aortic VSMCs by liposomes. Transfection of tRF-Gly-GCC had no significant effect on FMOD and HSP20 levels in VSMCs. **B** According to bioinformatics analysis, tRF-Glu-CTC may be a negative regulator of FMOD in VSMCs. After the transfection of tRF-Glu-CTC analogs into VSMCs, the level of FMOD was reduced.

## Data Availability

The datasets used and/or analyzed during the current study are available from the corresponding author upon reasonable request.

## Abbreviations

CPM: Reads per million
DE-tsRNAs: Differentially expressed tsRNAs
ELISA: Enzyme-linked immunosorbent assay
FMOD: Fibromodulin
GEO: Gene Expression Omnibus
LFQ: Label-free quantification
lncRNA: Long non-coding RNA
ncRNAs: Non-coding ribonucleic acids
NIH: Neointimal hyperplasia
miRNA: MicroRNA
RT-PCR: Reverse transcription-polymerase chain reaction
SD rat: Sprague–Dawley rat
SEM: Standard error of the mean
TGF-β1: Transforming growth factor-β1
tRF-Gln-CTG: TRNA-Gln-CTG-derived fragment
tRFs: TRNA-derived fragments
tsRNA: TRNA-derived small RNA
VSMCs: Vascular smooth muscle cells
