# Supplementary material for: Transfer RNA-derived small RNA tRF-Glu-CTC attenuates neointimal formation via inhibition of fibromodulin
Source: Cell Mol Biol Lett. 2024 Jan 3;29:2. doi: 10.1186/s11658-023-00523-z (PMC10763295; doi:10.1186/s11658-023-00523-z)
Supplement: Supplementary file 1 — Additional file 1. Supplementary Figure 1. ELISA showed that tRF-Glu-CTC inhibited fibromodulin (FMOD) levels in vascular smooth muscle cells (VSMCs). A According to bioinformatics analysis, tRF-Gly-GCC may be a negative regulator of fibromodulin (FMOD) and/or HSP20 (HSPB6) in VSMCs. Therefore, polynucleotide analogs of tRF-Gly-GCC were synthesized and transfected into rat thoracic aortic VSMCs by liposomes. Transfection of tRF-Gly-GCC had no significant effect on FMOD and HSP20 levels in VSMCs. B According to bioinformatics analysis, tRF-Glu-CTC may be a negative regulator of FMOD in VSMCs. After the transfection of tRF-Glu-CTC analogs into VSMCs, the level of FMOD was reduced. [file 11658_2023_523_MOESM1_ESM.docx]

**Supplementary Materials**


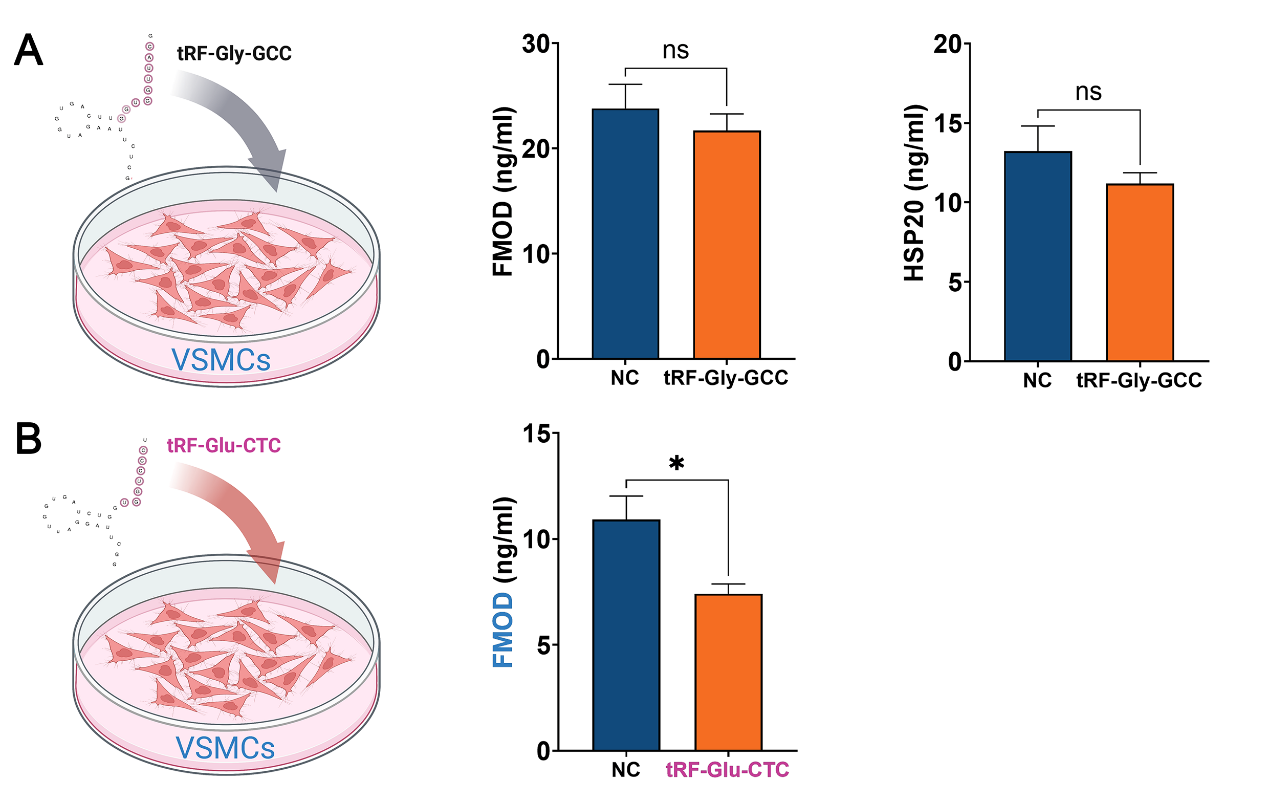


**Supplementary Figure 1.** ELISA showed that tRF-Glu-CTC inhibited fibromodulin (FMOD) levels in vascular smooth muscle cells (VSMCs). **A.** According to bioinformatics analysis, tRF-Gly-GCC may be a negative regulator of fibromodulin (FMOD) and/or HSP20 (HSPB6) in VSMCs. Therefore, polynucleotide analogs of tRF-Gly-GCC were synthesized and transfected into rat thoracic aortic VSMCs by liposomes. Transfection of tRF-Gly-GCC had no significant effect on FMOD and HSP20 levels in VSMCs. **B.** According to bioinformatics analysis, tRF-Glu-CTC may be a negative regulator of FMOD in VSMCs. After the transfection of tRF-Glu-CTC analogs into VSMCs, the level of FMOD was reduced.
